# Advocacy for active transport: advocate and city council perspectives

**DOI:** 10.1186/1479-5868-7-5

**Published:** 2010-01-24

**Authors:** Rosalina Richards, Linda Murdoch, Anthony I Reeder, Marieah Rosenby

**Affiliations:** 1 Cancer Society Social and Behavioural Research in Cancer Unit, Department of Preventive and Social Medicine, Dunedin School of Medicine, University of Otago, PO Box 913, Dunedin, New Zealand

## Abstract

**Background:**

Effective advocacy is an important part of efforts to increase population participation in physical activity. Research about effective health advocacy is scarce, however, the health sector can learn from the experiences and knowledge of community advocates and those who are on the receiving end of this advocacy. The aim of this study is to explore advocacy for active transport from the perspectives of community advocates and representatives from City councils.

**Methods:**

Cycling and walking advocates were identified from the local contact list of Cycling Advocates Network and Living Streets Aotearoa. Semi-structured telephone interviews were conducted with cycle and walking advocates from throughout New Zealand. Advocates also nominated a suitable council officer at their local City council to be interviewed. Interviews were recorded and transcribed and categories of responses for each of the questions created.

**Results:**

Several processes were used by advocates to engage with council staff, including formal council submissions, meetings, stakeholder forums and partnership in running community events promoting active transport. Several other agencies were identified as being influential for active transport, some as potential coalition partners and others as potential adversaries. Barriers to improving conditions for active transport included a lack of funding, a lack of will-power among either council staff or councillors, limited council staff capacity (time or training) and a culture of providing infrastructure for motor vehicles instead of people. Several suggestions were made about how the health sector could contribute to advocacy efforts, including encouraging political commitment, engaging the media, communicating the potential health benefits of active transport to the general public and being role models in terms of personal travel mode choice and having workplaces that support participation in active transport.

**Conclusions:**

There is potential for the health sector to make an important contribution to advocacy for active transport in New Zealand. While there are many barriers to achieving supportive environments for cycling and walking, a range of advocacy strategies were identified which could help ensure that health perspectives are considered in decisions relevant to active transport.

## Background

Effective advocacy is a priority in efforts to increase population participation in physical activity [1]. Defined as a *'combination of individual and social actions designed to gain political commitment, social acceptance, and supportive policy and systems' *[2], advocacy is a central component of health promotion [3] and important to the successful translation of research findings into evidence-based policy and practice [4].

The 'chaotic reality' of advocacy, involving a myriad of influences and opportunistic responses, is an uneasy fit with traditional research methodologies [5]. As a result, many factors underpinning the effective practice of advocacy remain relatively undocumented [5,6]. It is, therefore, vital that the health sector shares information about advocacy experiences, successes and failures so that practice can be refined and extended and outcomes improved.

An important step in this process has been the identification of a suite of advocacy strategies relevant to advancing the physical activity agenda [1]. These include; winning *political commitment *for physical activity initiatives, *media coverage *to mobilise public and stakeholder support, and *professional mobilisation *of the physical activity workforce to advocate for desired changes. Also important is the need for *community mobilization *to empower community members to advocate for their own needs and *advocacy from within *organisations to reorient policies, structures and programmes to support physical activity.

These strategies can be approached in a variety of ways, depending on the type of physical activity of interest and the intended target audience for advocacy activities. The focus of this paper is on a subset of overall physical activity participation, active transport. Active transport is associated with lower all cause mortality [7], increased fitness, decreased body weight and diastolic blood pressure among adults [8,9], and with greater physical activity among children [10]. One of the key audiences for active transport advocacy is local government. Local government plays a pivotal role in the creation of transport environments that either support or impede active transport. In New Zealand, there are 12 Regional councils, 16 City councils, and 57 District councils. Some of the key functions of City councils include community well-being and development, roading and transport infrastructure and recreation and culture within their city [11].

Evidence based public health is a relatively new voice in transport and urban planning [12,13]. However, there is potential for strong partnerships between the health sector and local government [14] and for health issues to have an impact on the transportation agenda [12,13]. It is important, however, that health advocates understand the competing priorities faced by transport professionals who must consider the needs of all road users and have to adhere to guidelines which may not allow them to incorporate health implications in decision-making [12].

As in many other countries, New Zealand has existing advocacy groups that are working to improve conditions for cyclists and pedestrians. The Cycling Advocates Network [15] and Living Streets Aotearoa [16] operate as national umbrella organisations supporting a network of locally based advocate groups that are active in their city, district or region. These agencies represent a significant pool of knowledge and experience about advocacy for cycling and walking and working with local government.

The aim of the current study was to explore current approaches to advocacy for cycling and walking from the perspective of community advocates and City council officers. Telephone interviews were used to explore how these individuals and agencies work together, perceived barriers to achieving advocacy goals and the other agencies that are relevant to active transport advocacy. Also examined was the use of research evidence in advocacy, best practice advocacy activities, and how the health sector can undertake or support active transport advocacy in New Zealand cities.

## Methods

### Participants

Local advocate groups from cities and towns throughout New Zealand were identified from the website contact list of the Cycling Advocates Network and Living Streets Aotearoa. Telephone interviews were conducted with representatives from 15 of the 20 existing cycle groups and 8 of the 9 walking groups. Advocates who were based in cities were asked to nominate a city council staff member who was their main contact within council. In cities where there was no advocate group (3 for walking and 1 for cycling) the customer services division of the council was contacted and asked who would be the person with responsibility for cycling or walking issues in their city. In total 28 interviews with council representatives were conducted (14 for cycling and 14 for walking, of a total of 16 councils). For 12 of the 14 council respondents, the same person was nominated to complete both the cycling and walking interviews.

### Measures

Respondents participated in a semi-structured telephone interview which took 20-60 minutes to complete. Prior to going to field, comment on the interview questions was sought from National Office staff of Living Streets Aotearoa and Cycling Advocates Network to ensure that questions were relevant and phrased appropriately. Included in the *interview for advocates *were questions about their advocacy group, motivations for involvement, how their group works with council, barriers for council in meeting their requests, other influential agencies in their city, their use of research evidence, evidence gaps, and ideas for how the health sector could get involved in advocacy. The *interview for City council staff *included questions about respondent's training and role in council, how their council works with community groups, barriers to meeting community concerns related to active transport, other influential agencies in their city, gaps in the research evidence, effective advocacy strategies and ideas for how the health sector can get involved in advocacy. The interview questions were open-ended and interviewers were able to prompt respondents for more information about specific issues mentioned. Interview questions were also able to be asked in a different order depending on the flow of the interview.

### Procedures

Respondents were sent an introductory email asking them to participate in the study with an information sheet attached that outlined the aim and procedures of the study. Contact was then made to arrange a suitable interview time. Confidential semi-structured telephone interviews were carried out from September to October 2008 by two of the authors (MR and RR). Ethical approval was given for the study from the Department of Preventive and Social Medicine, following University of Otago procedures.

### Analyses

With respondents' permission, audio recordings were made of all interviews, which were then transcribed for analysis. As this was an exploratory study, categories of responses to each question were created (LM and RR), with the most common reported here, along with illustrative quotes as appropriate.

## Results

### Respondents: Advocate groups and respondents

The membership and structures of advocacy groups varied around New Zealand. Memberships ranged from a handful to hundreds, with some having formal paid memberships and others being a more informal group. A commonly reported arrangement was a core group of active advocates and wider mailing lists of supporters and other interested individuals. Groups also varied in the time they had been operational. Some had existed for less than 1 year and others had been active for well over a decade, with pedestrian advocate groups tending to be newer than cycling ones. Another area of difference were the geographic areas that groups covered in their advocacy activities. These varied from discrete urban city areas to catchments including extensive rural areas or multiple cities. The pressure of wide catchments but limited people-power to advocate was raised by several respondents.

Advocacy in general is hard work, because advocacy is usually performed by amateurs in their spare time and often in organisations the burden often falls on just a few members of the group and its actually very very easy for those active members of the group to get burnt out. [Respondent 42, cycle advocate]

Advocates were asked about their motivations for being an advocate and those of others in their groups. A common response was a love of walking or cycling and, in turn, the desire to make this an easier transport choice. Other motivators included a concern for environmental sustainability and the relationship between active transport and health. The latter included both personal experiences of ill health or benefits from active transport as well as more general population health concerns.

"We are making our country sick, by making roads that you can't ride your bikes on" [Respondent 23, cycling advocate]

"A lot of the people who are involved in the group actually experience this kind of horrible dilemma. They would like to be more active, they'd like their families to be more active, but it's actually dangerous." [Respondent 43, pedestrian advocate]

Other respondents reported that a particular issue in their city had been the catalyst for getting involved in advocacy, while for others it had been the experience of travelling overseas and seeing other places which were perceived as being significantly more supportive of active transport modes.

### Respondents: Council officers

City council officers were asked about their current role at the council and their background and training for this position. Most respondents had responsibilities for transport planning and/or road safety and accordingly, reported backgrounds in engineering, but other training also included nursing, psychology, education, geography, parks and recreation and tourism management.

### Processes for working together

Formal council procedures were frequently used by advocates, such as written and verbal submissions to annual plans, cycling/walking policies and strategy documents. In addition, respondents reported less formal engagement such as regular meetings or email contact and collaboration on community events that promoted walking and cycling in their communities. Advocates also took part in 'key stakeholder' groups and forums related to broader issues of transportation or sustainability. There was a range of opinion about how advocates thought their feedback was received during these processes. On the one hand:

"Our experience in recent years has been that they've been very receptive and they've listened carefully, and in fact sometimes we've criticised certain aspects and the design has actually been changed as a response to our feedback" [Respondent 22, cycle advocate]

Whereas, on the other hand.

"It sometimes feels like you've had no impact at all, and you've been listened to politely then completely ignored... that can be very discouraging" [Respondent 45, pedestrian advocate]

Another important role that some advocate groups were undertaking was auditing and monitoring of the general cycling and pedestrian environment, and providing comment on plans for specific changes in the road network.

They're [advocates] our eyes and ears out there in the community and we act on their concerns. Usually - I mean sometimes there are reasons why we can't change things. But in terms of maintenance, you know if they bring to our attention where some trees need pruning or a pothole needs filling, then we have a good relationship with them. [Respondent 9, Council officer]

### Council barriers

Advocates and council staff gave very similar responses when asked about barriers to responding to advocate concerns. These included funding, long lead-in times for roading projects, political will-power, latent demand, staff capacity, road building culture. These will be discussed in more detail here.

A key issue was the availability of funding for implementing cycling policies or plans. While a 50% subsidy is available from the New Zealand Transport Agency (NZTA) to support cycling and walking projects, for some councils the costs of some projects were still too high. The potential for an increase in rates (fees paid by homeowners to local government to help pay for local services such as water, sewerage and rubbish collection) was seen as a very difficult issue politically.

We've got to recognise that the government subsidy has a lot to do with what we build. ... government will say "but we're putting more and more money into cycling, we agree, but its in the subsidy situation and that means that council has to fill in the other portion of the subsidy [Respondent 6, Council officer]

Also mentioned by many was the long lead-in time for many of the projects, with a lengthy cost benefit process needed to access the NZTA subsidy, and road upgrade and maintenance projects tending to be carried out over many years.

The programme for implementing our network is over many many years so we're unable to meet a great number of the aspirations that people come to us with in a time they consider satisfactory. [Respondent 8, Council officer]

Another politically sensitive issue was that of removing parking spaces to allow room for cycle lanes. When this was the case, significant opposition was sometimes raised by residents and businesses in the vicinity. Other competing interests were also identified by advocates, such as a powerful car lobby, freight companies and groups arguing for less local government spending. Council officers take into account feedback from all of these stakeholders, as well as from active transport advocates and other divisions of council.

We have to balance everything up and make decisions that are going to make the biggest impact for the largest number of people [Respondent 5, Council officer]

Related to this is the issue of latent demand. There may be a perception that, because there is no current demand, a facility is not needed. In the case of the creation of part of a cycle network, no increase may be seen directly after implementation, and may not be evidenced until the whole network is completed.

Another important issue raised was a lack of political will for making changes. The nature of this differed among councils with some having supportive (publically elected) councillors but unsupportive council staff while others reported the opposite situation.

" [council staff] have been very sympathetic towards pedestrian issues. Not quite so sure if all the [elected] members of council get it" [Respondent 42, pedestrian advocate]

" we're ok with the councillors... we have good relationships with the kind of middle management people... but it's the senior management... though they seem to be taking it a bit more seriously" [Respondent 21, cycling advocate]

Another significant issue, identified by both advocates and council staff was the capacity of council staff, in terms of expertise in design for cyclists and pedestrians, too few staff hours available and high staff turn-over.

"They come, they're very overworked and they don't stay very long and they go..." [Respondent 33, cycling advocate]

Several respondents also identified the presence of a road-building culture in New Zealand, which revolves around the needs of motorised transport. In addition, there was a perception that, since cyclists and pedestrians do not pay road-user charges, they were considered less important.

"The perception has been that... our roads are for cars and what we do is plan for vehicles and not really planning for moving people." [Respondent 11, Council officer]

"Cyclists want everything, pay nothing and choose not to obey the rules unless it suits them." [Respondent 6, Council officer]

### Other stakeholders

Respondents were asked to identify other council divisions, government agencies and community groups with an interest in cycling and walking initiatives.

As shown in Table 1, many council divisions were identified as being relevant and involved in decisions related to cycling and walking. A major external influence, mentioned by all respondents was the NZTA, the Government agency with responsibilities which include land transport planning, managing the state highway system, promotion of land transport safety and sustainability and allocation of government funding for land transport [17].

**Table 1:** Other agencies involved in cycling and walking advocacy in NZ cities

*City Council divisions*	
Transportation planning	Architecture
Road safety	Urban design
City planning	Leisure and recreation
Public transport	Parks and reserves
*Government agencies*	
Regional councils	District councils
Accident Compensation Corporation	New Zealand Transport Agency
Energy Efficiency and Conservation Authority	Ministry for the Environment
Police	Schools
District health Boards	Sport and Recreation New Zealand
Regional Sports Trusts	Department of Conservation
*Community groups, business and NGO's*	
Lions club/Rotary	Business Associations
New Zealand Recreation Association	Age Concern
Community boards	Iwi groups
Disability groups	Plunket
Automobile association	Freight companies
Recreational/racing cyclist groups	Recreational space lobbyists
University	Local Health Centres/GP's

Many other different government, non-government, and community organisations were identified, including several from the health sector. While there are those that would be natural coalition partners with health (e.g. schools, Regional Sports Trusts), also included are some agencies which may oppose some active transport initiatives, such as the car lobby (e.g. Automobile Association), freight companies and business associations.

### Use of research evidence in advocacy

Several advocates reported that they used research evidence in their advocacy activities, in particular, information about the design standards for cycling and walking facilities, economic benefits of active transport, and statistics from NZTA and councils themselves. Living Streets Aotearoa and Cycling Advocates Network were mentioned by many as having a key role in the summarising and distribution of research to local groups. The importance of having visiting international experts was also mentioned by many respondents. Nevertheless, while advocates themselves were interested in the research evidence, some advocates expressed doubt about the usefulness of this information as an advocacy tool.

"I'm of the view that many of our decision-makers in the past, and perhaps even currently, don't bother too much about evidence. They make up their mind on some other basis" [Respondent 45, pedestrian advocate]

Advocates and council staff were also asked about gaps in the research evidence. Both groups of respondents wanted information about barriers to cycling and walking, how far people are prepared to travel in these modes, deficiencies in existing infrastructure that inhibit cycling and walking and how to estimate latent demand for a proposed route or facility. Also of interest was the safety of cycle lanes, pedestrian perceptions of safety, economic benefits of active transport and accurate measurement of cycling and walking participation and injury.

### Council perspectives on effective advocacy

Council respondents were asked to describe the types of advocacy that work best from their perspective. Some of these responses related to advocate group structures and processes, including the benefits of having a formalised group, one representative to communicate with council, regular meetings with council to maintain a relationship, discussion/prioritisation of complaints and using formal submissions processes to comment on council activities. Also mentioned was the importance of building support among local politicians and community board members and generating media exposure for these issues.

"Relationships that I see working better are when they're more oriented towards building a broader public support, you know, saying we'll organise this forum and fight the media to show how much we care about this issue [Respondent 13, Council officer]

Other valued activities reported by council officers were partnerships on community events/promotions for walking and cycling and assistance with auditing for council projects.

They're asking the hard questions of the planning departments and the engineering departments - and that's a good thing [Respondent 9, Council officer]

More general comments about effective advocacy included having people who are passionate about the issue, being seen on your bike, and maintaining a positive focus. It was also suggested that advocates remain aware of the long time-frame for change and that council must also work for other residents/road users. Furthermore, it was suggested that excessive aggression and adversarial relationships may be counter-productive.

"Um well too many brickbats don't work, because it's all about maintaining a relationship, and if all we ever hear from these groups is, you know gripes and groans and we can never get it right, then you damage a relationship and those groups can sometimes isolate themselves." [Respondent 9, Council officer]

"I've sometimes thought that, you know, I don't actually need to have them annoying me when I'm working as hard as I can to achieve things in a system that's not necessarily that supportive." [Respondent 3, Council officer]

### Advice for how the Health sector can contribute to active transport advocacy

Advocates and council staff were asked to suggest ways that the health sector could contribute to advocacy for active transport. Respondents suggested that an important role is to promote the benefits of cycling and walking. This included messages targeted at the general public and engagement of the media to highlight some of these issues.

The direct involvement from the District Health Board would be really useful, and specifically in terms of making people aware of the obesity issues and the health benefits of cycling, that we can allude to - but we're not really qualified to put out to the public domain. [Respondent 5, Council officer]

Someone needs to be devising national ads that are targeting the benefits of cycling and walking in terms of health and the carbon footprint. [Respondent 7, Council officer]

Another common suggestion was involvement in formal council processes (such as submissions to Annual Plans and Long Term Community Consultation Plans), which would give health sector the opportunity to present these arguments to council staff and elected councillors. This was seen as particularly helpful because of the respect given by council for the views of health professionals.

"They kind of think, well we don't need to worry about cycling and walking... OK there's some congestion, but that's nothing that a few bigger roads won't fix up. But when you talk about the health implications of cancer or heart disease ... then that just throws them completely... so its helpful" [Respondent 20, cycling advocate]

"I see health as the key to unlocking most of the doors to this, to get in the changes" [Respondent 21, cycling advocate]

Interestingly there was also a caution about an overemphasis on injury and safety over messages about potential health benefits that can be accessed from active transport.

"Unfortunately my view of what comes out of these [health]... groups tends to be very much injury and safety focussed" [Respondent 11, cycling advocate]

"To me it doesn't matter, um that sounds harsh, but we shouldn't be so fixated on the injuries as part of the cost-benefit analysis, we should be much more focused on the benefits - in health terms... I don't think that adequate consideration is given to the health side of it [Respondent 12, Council officer]

Other suggestions were to get to know local advocates and council staff, assisting at community promotions of active transport and for individuals to become an active commuter. It was also suggested that health employees could undertake some advocacy within their own workplace to ensure that cycling and walking are viable transport modes for individuals working in and visiting health sector organisations. Finally, another important role identified was to support community mobilisation, in terms of offering encouragement and support for those volunteers who already advocate for cycling and walking in their community.

To have health people making just a nice clear kind of statement about why this cycling project is really important is so helpful, not just on making the argument convincing but, as I say, really invigorating for advocates - who are themselves at risk of burnout." [Respondent 20, cycle advocate]

## Discussion

Effective advocacy is an important part of efforts to increase physical activity participation overall [1] and for particular components of activity such as active transport. The health sector is well poised to make a contribution to advocacy for active transport, as individuals and agencies have a strong presence and extensive networks within their local communities. The focus of this study was a specific aspect of advocacy, advocacy for cycling and walking to a local government audience, and draws from the experiences of the advocates and council staff who work at the coal face of this issue.

Consistent with previous findings [14], the current study found significant potential for partnership between the health sector and local government. Identified here are several processes that could be used by the health sector to engage with city councils, ranging from formal submissions to collaboration on initiatives to promote active transport. An important aspect of potential partnerships is an understanding of the tensions experienced by council staff related to active transport issues [12]. Several tensions were highlighted by respondents here, including a lack of funds for cycling and walking initiatives, variable political support for change and limitations in the staff expertise and time available to address active transport issues.

Also identified here are potential candidates for coalitions to support active transport. Collaboration and alliance building are an important part of effective advocacy [18] and there is potential for strategic alliances between sectors such as environmental sustainability and obesity [19]. Some potential opponents for efforts to increase active transport were also discussed here. Previous work has described some generic 'enemies' for physical activity such as labour saving devices and apathy [1]. In the case of active transport, there appear to be those with more concrete vested interests who will respond to proposed changes that pose a threat or inconvenience to them. These threats may include rates rises, removal of parking spaces, traffic calming or other actions that challenge the prioritisation of motorised traffic over other modes. The identification of opposing arguments is an important task, allowing advocates to frame messages to refute these [5].

Encouragingly, the advocacy strategies that were highlighted by active transport advocates and council staff 'on the ground' were very consistent with those that have been identified as being key for advancing physical activity generally [1]. Respondents highlighted activities such as submissions to council annual plans to build *political commitment*. The need for *media coverage *was also mentioned, in order to gain political attentions, and for *community mobilisation *via communication of health benefits to the general public and raising awareness of active transport concerns. An additional role in community mobilisation is offering support and encouragement to existing cycling and walking advocate groups who may be struggling because of member stress and burn-out. *Advocacy from within organisations *was also raised by respondents as an important advocacy strategy. Health sector organisations are large employers and can act as a role model in encouraging and supporting staff to participate in active transport. Linked to this is *professional mobilisation *to empower individuals and agencies within the health sector to advocate for active transport. Respondents were very positive about the potential gains from greater involvement from the health sector and it is hoped that this study can help facilitate some of this work.

There are some limitations to this study that need to be mentioned. The first is the selection process used for advocates and council respondents. Advocates were identified from a list of contacts for local advocate groups rather than a random sample of all advocate members. As views may differ between group members, the responses reported here are a series of advocate perspectives rather than representative of all advocate views. Similarly, for council respondents, a different sampling approach, for example targeting councillors, senior transportation managers or community and recreation staff may give different perspectives, which is an interesting issue for further study. Other future directions for study were identified by respondents including barriers to participation in active transport, potential distances that could be travelled, measurement of active transport and estimation of accurate injury statistics. In terms of advocacy research, there is a need for further research that describes and evaluates advocacy efforts by the health sector. This study has focused on local government, however, work with central government is also vital. Given the importance of advocacy for advancing health outcomes, the health sector urgently needs to build its capacity in this area.

## Conclusions

There is potential for the health sector to make an important contribution to advocacy for active transport in New Zealand. There are a number of processes identified here that could be used to engage with city councils, as well as potential allies for these efforts. While there are many barriers to achieving supportive environments for cycling and walking, a range of advocacy strategies were identified here that could help ensure that health perspectives are considered in decisions relevant to active transport.

## Competing interests

The authors declare that they have no competing interests.

## Authors' contributions

RR conceived of the study, participated in design, data collection, data analysis and drafted the manuscript. AIR participated in the design of the study and helped in drafting the manuscript. MR participated in the design, coordination and data collection, LM analysed the data and helped draft the manuscript. All authors read and approved the final manuscript.
